# Use of placebo interventions among Swiss primary care providers

**DOI:** 10.1186/1472-6963-9-144

**Published:** 2009-08-10

**Authors:** Margrit Fässler, Markus Gnädinger, Thomas Rosemann, Nikola Biller-Andorno

**Affiliations:** 1 Institute of Biomedical Ethics, University of Zurich, Zurich, Switzerland; 2 Department of General Practice, University Hospital Zurich, Zurich, Switzerland

## Abstract

**Background:**

Placebo interventions can have meaningful effects for patients. However, little is known about the circumstances of their use in clinical practice. We aimed to investigate to what extent and in which way Swiss primary care providers use placebo interventions. Furthermore we explored their ideas about the ethical and legal issues involved.

**Methods:**

599 questionnaires were sent to general practitioners (GPs) and paediatricians in private practice in the Canton of Zurich in Switzerland. To allow for subgroup analysis GPs in urban, suburban, and rural areas as well as paediatricians were selected in an even ratio.

**Results:**

233 questionnaires were completed (response rate 47%). 28% of participants reported that they never used placebo interventions. More participants used impure placebos therapeutically than pure placebos (57% versus 17%, McNemar's χ^2 ^= 78, p < 0.001). There is not one clear main reason for placebo prescription. Placebo use was communicated to patients mostly as being "a drug or a therapy" (64%). The most frequently chosen ethical premise was that they "can be used as long as the physician and the patient work together in partnership" (60% for pure and 75% for impure placebos, McNemar's χ^2 ^= 12, p < 0.001). A considerable number of participants (11–38%) were indecisive about statements regarding the ethical and legal legitimacy of using placebos.

**Conclusion:**

The data obtained from Swiss primary care providers reflect a broad variety of views about placebo interventions as well as a widespread uncertainty regarding their legitimacy. Primary care providers seem to preferentially use impure as compared to pure placebos in their daily practice. An intense debate is required on appropriate standards regarding the clinical use of placebo interventions among medical professionals.

## Background

Numerous studies demonstrate that placebo interventions can have an influence on outcomes and may benefit the patient [1-5]. On the other hand, the evidence that placebo interventions can significantly alter clinical outcomes has been questioned, and some authors have cautioned against their possible negative effects [6-8]. This unsettled dispute about the appropriate role of placebo interventions in clinical settings is reflected by the regulatory situation: While the use of placebos in research is extensively regulated by national and international guidelines, their use in clinical practice often occurs in a legal and ethical gray zone. In this context of normative uncertainty the standards that primary care providers develop to guide their own practice are of particular interest.

Various definitions of the term "placebo" have been suggested in the literature. We usually prefer the term "placebo intervention" because it is not only the concrete placebo "vehicle" (i.e. the drug or the procedure) that can influence outcomes but rather all context factors including the perceptions and expectations of the patients. We define a placebo intervention as an intervention which has no direct pharmacological, biochemical or physical mechanism of action according to the current standard of knowledge. It can take the form of a placebo medication or of a diagnostic or therapeutic sham procedure.

We chose to use the distinction between "pure" and "impure" placebos, which is well established in the literature [6,8-10]. "Pure placebos" refer to inert substances or methods, whereas "impure placebos" include substances or methods which do have a known pharmacological or physical activity but which cannot be expected to have any direct therapeutic effects for the respective disease and in the chosen dosage.

We decided to use this distinction although it cannot be applied to all placebo interventions in a clear cut way – for instance, diagnostic placebo interventions cannot easily be attributed to either category. Our hypothesis was that clinical use and the moral reasoning accompanying it would be quite different for both categories so that the distinction would help us to gain a more nuanced understanding of the ways physicians think about and use placebo interventions.

In the past twenty years, six surveys have been published that address the attitudes and habits of physicians with regard to the use of placebo interventions [10-15]. The reported percentage of physicians using placebo interventions in clinical practice ranged from 41% to 99% depending on their specialty and, more importantly, the context of the survey question. In a Swedish study for instance, the use of antibiotics for viral infections was presented to the physicians as a placebo treatment. Thus the physicians were focused on *impure placebos *when they were asked about their prescribing habits regarding placebo treatment. This may have led to the high percentage of positive responses (99%) [15]. There are publications of surveys which do not even mention if a certain definition of the term "placebo" was given to participants [13,15]. Defining the term is highly important, however, because many definitions and conceptions of placebo exist in the literature and therefore different conceptions of placebos among survey participants will influence their statements.

Until now no survey has explored whether physicians handle pure and impure placebos differently, and the reasons for rejecting or accepting the use of placebo interventions have only been touched upon. To our knowledge ours is the first survey which systematically studies the potential differences in the use of pure as compared with impure placebos. The overall goal of our study was to determine to what extent and in which ways Swiss primary care providers utilise placebo interventions. Furthermore we explored their ideas about the ethical and legal issues involved.

## Methods

### Participants

In Switzerland, primary care is mainly provided by general practitioners (GPs) and paediatricians, so we chose to send our questionnaire to those two groups. Postal addresses were received from a register of the medical association of the Canton of Zurich and additionally from the database . A total of 149 addresses of paediatricians and 1518 of general practitioners in private practice were available. We stratified the addresses of the GPs according to their location and selected randomly 150 GPs from urban areas, 150 GPs from suburban areas, and 150 GPs from rural areas. The groups were coded by using different colours for the questionnaires. The group size was determined by the number of available paediatricians. Overall we included 599 primary care providers in our survey.

The Research Ethics Committee of the Canton of Zurich stated that the study did not require ethical review.

### Questionnaire

The questionnaire consisted of 14 multi-unit questions on four pages and can be downloaded from  in its original German version. For an English version see the Additional file 1. Reponses to questions no. 11–14, which focus on participants' ideas about the mode of action of placebo interventions and their relationship to complementary medicine, will be reported and discussed elsewhere.

On the top of the questionnaire, we placed our definitions of "placebo intervention", "pure", and "impure placebos" (Table 1).

**Table 1:** Definition of Placebo Intervention, Pure, and Impure Placebos

**Placebo intervention**	We define a placebo intervention as a diagnostic or therapeutic sham intervention or as an intervention with substances or physical methods which have no direct pharmacological, biochemical or physical mechanism of action according to the current standard of knowledge. The term includes a considerable variety of interventions, thus not only the administration of lactose tablets or isotonic saline solution.
**Pure placebos**	Are inert substances or methods such as sugar pills or isotonic saline solution.

**Impure placebo**s	Refer to substances or methods which have a known pharmacological or physical activity but which cannot be expected to have any direct therapeutic effects for the respective disease and in the chosen dosage, e.g. vitamin infusions for cancer or peppermint pills for pharyngitis.

Subsequently the questionnaire pointed out that the questions referred only to the use of placebo interventions in clinical practice, not to placebos given as controls in clinical trials.

In addition to demographic data we focused on the following questions about the use of placebo interventions: Which placebo interventions study participants use, whether they use placebo interventions at all, for which motives, how often, what they communicate to the patient, their attitude towards placebos, and the estimated disappointment of patients were they to discover that they had unknowingly received a placebo. Responses required the distinction between pure and impure placebos.

In three consecutive pilot studies, we pretested our questionnaire with a total of 27 GPs or paediatricians and revised some wording. The survey was conducted between December 2007 and March 2008. The questionnaires were sent out by mail with a letter explaining the aim of the survey and the anonymous data processing. Two reminders were sent out. Codes on the returning envelopes were used to identify participants who did not need a reminder. On return, the envelope was separated from the questionnaire for anonymous data processing.

### Statistical analysis

We processed our data analysis using SPSS version 16. Every item was checked for differences caused by the following variables: location area (urban vs.

suburban vs. rural), paediatricians vs. GPs, gender and age group (<50 vs. ≥ 50 yrs). In order to reduce multiple statistical testing and to obtain only relevant group differences, we first checked for group differences amounting to over 15%, and applied χ^2^-testing only to items with group differences above the cut off.

Differences in ordinal scale variables were assessed by Wilcoxon's rank sum test (e.g. presumed disappointment after knowledge of having received pure as compared to impure placebo), while binomial variables were compared with McNemar's χ^2 ^test (e.g. consent to pure vs. impure placebo treatment). Statistical significance level was 0.05 with a two tailed hypothesis. Values are given as frequencies or mean and SD.

## Results

The final version of the questionnaire was sent out to 599 Swiss primary care providers who practise in medical offices. Two of them were returned because their addresses were unknown, and 15 respondents declared not to be a GP or paediatrician or not to have a private practice. This left a sample of 582.

The questionnaires were collected by MG who managed the reminders. For the extraction of data the questionnaires were sent from MG to MF as soon as a significant amount had been received. 83 of these, mostly filled questionnaires got lost by the mailing company and could not be retrieved. To calculate the response rate we subtracted the 83 lost questionnaires from the original sample of 582 and set 499 as 100%, which leads to a 47% response rate for the available 233 completed questionnaires.

### Demographics

The characteristics of the respondents are shown in Table 2. The return rate of the primary care providers in urban areas was lower than in suburban or rural areas. The proportion of female respondents (29%) corresponds to the percentage of women in private practice in primary care in the Canton of Zurich (22%) [16].

**Table 2:** Sample characteristics

**Category**	**N**	**Count (percentage) or mean ± SD**
Group of physicians	233	
Paediatricians		67 (29%)
GPs in urban areas		41 (18%)
GPs in suburban areas		55 (24%)
GPs in rural areas		70 (30%)
Gender	227	
Women		65 (29%)
Men		162 (71%)
Age (years)	228	52 + 9
Days per week working in private practice	197	4.1 + 1.2
Patients seen per day	224	
<15		35 (16%)
15–30		150 (67%)
>30		39 (17%)

### Use of placebo interventions

66 of the 233 (28%) participants reported that they did not make any clinical use whatsoever of interventions they considered a placebo. To answer the question whether the frequency of therapeutic use of pure and impure placebos was different we took the data from participants who answered both question 5 and 6 ("If you use pure/impure placebos therapeutically – what do you tell the patient?"), i.e. 203 of the 233 participants. Significantly more participants used impure placebos (116 of 203) than pure placebos (34 of 203) for therapeutic purposes (57% versus 17%, McNemar's χ^2 ^= 78, p < 0.001). 93% of those who do use pure placebos used them once a month or less, 2% weekly, and 5% daily.

Table 3 shows how often different placebo interventions are utilised by the participants. In the questionnaire respondents could indicate their view on the respective intervention being a placebo intervention or not (last two columns). The most frequently mentioned placebo interventions were diagnostic procedures in the form of non-essential physical examinations of the patient (89%), followed by positive suggestions (81%). Only 7% of the participants had provided sugar pills, 10% reported injections with saline solution. Again we did not find any group differences.

**Table 3:** Use of placebo interventions and the question whether the participants view the respective intervention as placebo intervention

	**N**	**. I have already used**	**. I have not used so far**	**N**	**... is not a placebo intervention**.
Positive suggestions	180	146 (81%)	34 (19%)	197	62 (32%)
Simple ointments and/or bandages for contusions without visible skin damages	181	137 (76%)	44 (24%)	197	52 (27%)
„Sugar pills”	196	14 (7%)	182 (93%)	197	3 (2%)
Injections with saline solution	198	20 (10%)	178 (90%)	199	4 (2%)
Therapies without pharmacological or physical efficacy for the patient's conditions (e.g. vitamins or antibiotics without approved indication)	195	119 (61%)	76 (39%)	196	12 (6%)
Diagnostic practices:					
• non-essential physical examinations of the patient	193	171 (89%)	22 (11%)	200	38 (19%)
• non-essential technical examinations of the patient without relevant risks (e.g. ultrasound, MRI)	195	134 (69%)	61 (31%)	197	24 (12%)
• non-essential technical examinations of the patient with relevant risks (e.g. computed tomography)	192	59 (31)%	133 (69%)	195	21 (11%)

### Motives for the use of placebo interventions

Table 4 shows the motives for using placebo interventions among the participants using placebos for diagnostic or therapeutic reasons (n = 167). Considering both, the group who uses just impure placebos and the group who uses both pure and impure placebos together, the most frequently chosen motives were "to gain therapeutic advantage through the placebo effect" (69%), "to offer a treatment to patients whose complaints and test results are not attributable to a certain disease" (64%), and "to conform with the requests of the patient" (63%).

**Table 4:** Motives for the use of placebo interventions among the 167 users

	**N**	**I use pure **and impure placebos*	I use only impure placebos*	**I use no placebos at all**
To conform with the requests of the patient	159	17 (11%)	83 (52%)	59 (37%)
To gain a therapeutic advantage through the placebo effect	157	31 (20%)	77 (49%)	49 (31%)
To still be able to offer a treatment option to a patient with an „incurable” disease	154	12 (8%)	55 (36%)	87 (56%)
To offer a treatment in situations in which standard treatments may strongly burden patients with side effects or are contraindicated	158	13 (8%)	46 (29%)	99 (63%)
To offer a treatment to patients whose complaints and test results are not attributable to a certain disease (unspecific complaints)	162	24 (15%)	80 (49%)	58 (36%)
To offer treatment to difficult patients with psychological peculiarities, i.e. constant unwarranted complaints	156	19 (12%)	61 (39%)	76 (49%)
To test whether the pain is psychogenic or organic	154	20 (13%)	12 (8%)	122 (79%)
To avoid drug addiction	155	12 (8%)	35 (23%)	108 (70%)

*Definitions see Table 1.

The total percentage may not equal 100 due to rounding.

The two least frequently chosen motives overall were "to test whether the pain is psychogenic or organic" (21%) and "to avoid drug addiction" (30%). All items were chosen more frequently by those who stated to use impure placebos only, with the exception of using placebos as a test to distinguish "psychogenic" from "organic pain". The participants had the possibility to note additional motives that guided their use of placebo interventions but only 4 of the respondents made use of this option.

When looking for interaction with our tested class variables, we found the following group differences: Fewer paediatricians than GPs used only impure placebos to treat patients with unspecific complaints (23 vs. 40%, p < 0.05). Also fewer paediatricians than GPs used only impure placebos in difficult patients (13 vs. 33%, p < 0.01).

### Communication about placebo use

When using pure or impure placebos, most participants reported telling their patients that they received a drug or a therapy (see Table 5). No one reported that he or she told the patients that the intervention was a placebo. Relatively few referred to the placebo as "a medicine with no specific effect" (11% for pure, 27% for impure placebo).

**Table 5:** What participants tell their patients when using placebos for therapeutic purposes

	**Use of pure placebos therapeutically (n = 222)**	**Use of impure placebos therapeutically (n = 208)**
*User:*	35	119*
I tell them		
... this is a drug/a therapy.	22 (63%)	77 (65%)*
... this is a placebo.	0 (0%)	0 (0%)
... this is a medicine with no specific effect.	4 (11%)	32 (27%)*
I say nothing.	9 (26%)	10 (8%)
*Nonuser:*		
I never use placebos for therapeutic purposes.	187	89*

Only one answer allowed, *p < 0.001 vs. pure placebo by McNemar's χ^2 ^test.

Percentages refer to the number of users.

### Ethical and legal issues of using placebo interventions

Study participants were asked if they agreed or disagreed with six statements about the use of pure or impure placebos in medical practice (Table 6). Their "I am uncertain" answers ranged from 11 to 38%, with the statement "The use of placebos must be rejected in principle because there are legal concerns" provoking most uncertainty (38% for the use of pure placebos, 30% for impure placebos). The most frequently confirmed statement for both pure and impure placebos was "The use of placebos can be used as long as the physician and the patient work together in partnership" (60% for pure vs. 75% for impure placebo). For all six questions, there was more agreement with the statements regarding the use of impure placebos than of pure placebos (Wilcoxon's rank sum test, z = 8.2, p < 0.001).

**Table 6:** Statements regarding the ethical and legal legitimacy of placebo use

		**Use of**	**pure**	**placebos**		**Use of**	**impure**	**placebos**	
	
	N	I agree	I am un-certain	I disagree	N	I agree	I am un-certain	I disagree	p value*
The use of placebos									
• must be rejected in principle because it is ineffective.	220	58 (26%)	37 (17%)	125 (57%)	220	41 (19%)	26 (12%)	153 (70%)	p < 0.05
• must be rejected in principle because it implies deceiving the patient.	223	101 (45%)	39 (17%)	83 (37%)	221	52 (24%)	39 (18%)	130 (59%)	p < 0.001
• must be rejected in principle because of legal concerns.	220	43 (20%)	84 (38%)	93 (42%)	221	30 (14%)	67 (30%)	124 (56%)	n.s.
• can be used as long as physician and patient work together in partnership.	220	132 (60%)	35 (16%)	53 (24%)	221	166 (75%)	25 (11%)	30 (14%)	p < 0.001
• is acceptable for the benefit of the patient and for minimizing harm to the patient.	219	120 (55%)	43 (20%)	56 (26%)	221	158 (71%)	24 (11%)	39 (18%)	p < 0.01
• is for me a traditional component of medical practice.	219	37 (17%)	25 (11%)	157 (72%)	217	91 (42%)	26 (12%)	100 (46%)	p < 0.001

Multiple answers allowed. * Significance testing by use of McNemar's Chisquare test, pure vs. impure placebo ("I am uncertain"-answers were excluded from analysis).

The total percentage may not equal 100 due to rounding.

As a group difference we found that paediatricians less often than GPs rejected the use of pure placebos for its implying the deception of patients (43 vs. 60%, χ^2 ^= 4.7, p = 0.03).

### Presumed patient reaction to being informed about placebo use

A considerable percentage of participants presumed that many of their patients would be disappointed if they came to know that they had been intentionally treated with a placebo: This worry was more pronounced regarding the use of pure placebos (55%) than in the case of an impure placebo (22%) (p < 0.001 by Wilcoxon's rank sum test) (Table 7). Less paediatricians than GPs were worried about their patient's disappointment when a pure placebo had been used (49 vs. 68%, χ2 = 12.4, p < 0.01).

**Table 7:** Presumed disappointment of patients if they learned that they had been intentionally treated with a pure or impure placebo*

	**Use of pure placebo (n = 223)**	**Use of impure placebo (n = 225)**
Yes, many of my patients	123 (55%)	49 (22%)
Yes, some of my patients	47 (21%)	53 (24%)
Mostly no	27 (12%)	103 (46%)
I do not know.	26 (12%)	20 (9%)

* Counts for pure vs. impure placebo were significantly different, Wilcoxon's, z = 8.7, p < 0.001.

## Discussion

Our survey gives a detailed picture of the use of placebo interventions in clinical practice and illustrates the attitudes of primary care providers. 72% of the 233 participants use placebo interventions in certain situations. Overall, far more participants make therapeutic use of impure placebos than of pure placebos (57% vs. 17%). This corresponds to results of a recently published US survey in which 41% of the physicians recommended over the counter analgesics as placebo treatment and 2–3% recommended inert substances within the past year [10]. Within our sample of primary care providers we could not detect any differences in the frequency of using pure placebos regarding their medical specialty (GP or paediatrician) or the localisation of their practice.

The most frequently used justification for the use of placebos in medical practice was that it can be used as long as the physician and the patient work together in partnership: 60% of all participants agreed with this statement with regard to the use of pure placebos, and 75% agreed with regard to impure placebos. This emphasis on a therapeutic partnership of patients and physicians contrasts, however, with participants' concern about their patients' disappointment if they found out they were intentionally treated with a placebo. Possibly physicians would want to find ways to use placebos in a non-deceptive manner that is in accord with a partnership model of the physician-patient-relationship, but have not yet found a suitable way of realizing this.

The most frequently endorsed argument against the use of placebos concerned the deception it implied vis-à-vis the patient: 45% of the participants agreed with this argument regarding pure placebos, and 24% regarding impure placebos (p < 0.001 for pure vs. impure placebos). Other surveys asked somewhat different questions. An Israeli survey reported that 5% of participants thought that the use of placebos should be categorically prohibited [13]. Surveys from the USA obtained 7 to 12% for this item [10,12], and a Danish survey found that placebo interventions were ethically unacceptable to 40% of study participants [11]. As the placebo definitions that the studies used differ, the possibilities to compare these data are limited.

Why is it useful to distinguish pure from impure placebos? The use of pure or inert placebos has met with scepticism by ethicists mainly because it presumably implies the deception of the patient, representing an outdated, unduly paternalistic physician-patient-relationship [17,18]. By contrast, the use of impure or active placebos seems easier to justify by the (to some extent sophistic) argument that, though not probable, these placebos could have some pharmacological or physical activity that has a positive effect on the health of the patient but is difficult to verify. One could also argue that interventions with impure placebos may have greater placebo effects than interventions with pure placebos because the theory and framework of the impure placebo intervention can lead to positive expectations or conditioned responses in patients. An intervention that is accompanied by positive information in the lay press can be expected to work better than a nameless pill without package insert. This assumption is in accordance with recent data showing that the use of expensive placebo pills reduces pain better than the use of cheaper ones [19]. On the other side it should not be neglected that impure placebos can cause harmful side effects, which incidentally can be severe, e.g. an allergic reaction to an antibiotic given for a viral infection.

In our sample, the use of placebos, be they pure or impure, was usually communicated to the patient as a "medication" or "therapy". It is unknown whether telling patients explicitly the nature of placebo treatment compromises placebo effects or not. The existing empirical data that deny an influence are weak [20]. It is possible that physicians underestimate the effectiveness of placebo interventions in adequately informed patients as well as the openness of the latter to try and take advantage of the power of the placebo. But this would need to be explored further in studies focusing on the patient's perspective. Still, physicians' information about medical interventions frequently include a grain of interpretation or untruthfulness – be it that the prospect of cure is somewhat exaggerated or the likelihood of adverse effects a bit downplayed. Determining what degree of such paternalistic, albeit benevolent, manipulation of medical information is appropriate for the individual patient-physician-relationship is part of a physician's responsibilities. Often there is an implicit mutual agreement and trust that the doctor will make the right choice of treatment.

A remarkable finding is the uncertainty among participants as regards the ethical and legal status of placebos as well as their effectiveness. 11 to 38% were uncertain how to appraise the respective statement presented to them in the questionnaire. Another cue revealing the uncertainness of participants is that we found only limited agreement on the motives to use placebo interventions. 72% of the participants administer placebo interventions to their patients in certain circumstances. There is less agreement among those who use placebo interventions for certain motives than might be expected. About two-thirds of those who use placebo interventions agreed with the three motives "to gain a therapeutic advantage of a placebo effect", "to offer a treatment to patients whose complaints and test results are not attributable to a certain disease", and "to conform with the requests of the patient".

Other authors found still less consensus regarding the motives or circumstances among placebo users. Sherman and Hickner reported that for the respondents who used placebos the most common motive was "to calm the patient" and "as supplemental treatment" (both 18%) [12]. In the survey of Nitzan and Lichtenberg the most frequently reported circumstance for placebo use was "after 'unjustified' demand for medication" (48%) [13]. Berger found 48% of those who used as well as those who usually did not use placebos were likely to use a placebo when they suspected factitious pain [14]. Only Hróbjartsson and Norup referred to a more stable consensus: 70% of the general practitioners who used placebo interventions agreed about the motive "following the wish of the patient and avoiding a conflict with the patient" [11]. All in all, the use of placebo interventions in general practice does not seem to follow clear patterns, in particular not from an international, cross-cultural perspective. There is not one main clear reason for placebo use.

The reason for the uncertainness of participants may well be due, at least in part, to their unfamiliarity with recent or even well-established findings of placebo research, e.g. as being reviewed by Price et al. [21]. Another reason may be that there is no commonly accepted consensus in the literature on how to use placebo interventions in clinical practice. We found neither (Swiss) national nor international policies which would help answering the question when and how to use placebo interventions in practice, although individual authors have made suggestions to that effect [22]. The ubiquitous requirement of informed consent is not fine-grained enough to provide much guidance when it comes to the more subtle issues of using placebo interventions. For example, there is no guidance on how to explain the mechanism of action of a proposed placebo intervention to the patient. Finding the right balance between putting the patient off by being too explicit about the placebo nature of the intervention and by being so vague as to be almost deceptive is a challenging task that might benefit from well-tailored recommendations.

A reassuring finding was that a vast majority of the Swiss primary care providers (85%) denied the factual use of placebos to test whether pain is psychogenic or organic. There is no evidence in the literature to corroborate the utility or plausibility of this test [14,23,24]. Ignoring the slightly different wording of questions in surveys addressing the usefulness of placebos to distinguish symptoms that have a psychogenic versus an organic origin, there is a high variability of adherence to this misleading theory among physicians: 19% [25], 20% [12], 53% [24], 68% [14] when asked for hypothetical use and 4% [12], 18% [11], 19% [13], 48% [14] when asked for the factual use.

Some limitations of our study need to be acknowledged. To illuminate many aspects of the use of placebo interventions in clinical practice, we developed a rather extensive questionnaire which may have cut the response rate. Non-respondents and missing data may have led to possible biases. Other surveys about placebo use also had response rates under 60% [10,12,23,26], which may well have its reasons in the somewhat delicate nature of the topic on the one hand and the high workloads of practicing physicians on the other, reducing the readiness to participate in research [27]. A formal testing of differences in age and gender for respondents and non-respondents cannot be presented for our study because we did not collect these data from non-respondents. Therefore we can not say whether the low response rate of our survey introduced marked bias to our data. However, the age and gender of the respondents do fit well with the data of Zurich Medical Association.

Despite our clearly stated definition of placebo interventions, the answers of the participants may have been influenced by different concepts about placebo interventions. For example, primary care providers who practice psychotherapy may have been confused our definition not explicitly excluding psychotherapy. Placebo effects can also be conceived as psychologically mediated effects and therefore it seemed inadequate and artificial to us to draw a sharp, explicit line between the effects of psychotherapy and placebo effects for the purposes of our definition. Still, had we explicitly included or excluded psychotherapy in our definition some participants might have replied differently to the question whether they do or do not apply placebo interventions to their patients.

The reported prevalence of use of pure placebos depended on the subjective criteria used to separate pure and impure placebos. For example, participants could consider homoeopathic remedies as pure placebos or not. Also, we did not ask for the frequency of the use of impure placebos because we assumed that answering this question would be very difficult as their use was too prevalent to be reliably quantified by an estimation. [15].

In clinical practice, physicians are often confronted with situations in which either a scientifically proven therapy is not known, may have unacceptable side effects, or in which the patient's complaints cannot be interpreted within the framework of a well-defined diagnosis. In such cases the physician could wait and see whether the "unspecific" symptoms may disappear without any intervention. But many patients do not get better by mere waiting, and their subjectively perceived illness remains considerable. Why not harness "the sometimes powerful beneficial effect of a placebo" [28]? The ambivalence and uncertainty of physicians who do not want to fall into the trap of arrogant and inappropriate paternalism is quite understandable. There may be ways, however, of getting the "power of the placebo" to work without losing the patient as a partner. Exploring those possibilities and defining suitable limits to the clinical use of placebo interventions seem to be an important next step [29].

## Conclusion

The data obtained from Swiss primary care providers reflect a broad variety of views about placebo interventions as well as a widespread uncertainty regarding their legitimacy. Primary care providers seem to preferentially use impure as compared to pure placebos in their daily practice.

An intense debate is required on appropriate standards on the clinical use of placebo interventions among medical professionals that reaches out to the perspective of those whose interests and wellbeing are the ultimate goal of all medical interventions: the patients.

## Competing interests

The authors declare that they have no competing interests.

## Authors' contributions

MF conceived the study. MF, NBA, and MG participated in the study design. MG performed the pilot studies of the questionnaire and mailed the final questionnaires. MF collected the data and MG analyzed them. All authors participated in the interpretation of results. MF wrote the first draft of the manuscript, and all authors critically reviewed it and contributed to subsequent drafts. All authors have seen and approved the final version of the manuscript.

## Pre-publication history

The pre-publication history for this paper can be accessed here:



## Supplementary Material

Additional file 1 **English version of the questionnaire.** Click here for file
