# Supplementary material for: Use of placebo interventions among Swiss primary care providers
Source: BMC Health Serv Res. 2009 Aug 10;9:144. doi: 10.1186/1472-6963-9-144 (PMC2731747; doi:10.1186/1472-6963-9-144)
Supplement: Additional file 1 — English version of the questionnaire. [file 1472-6963-9-144-S1.pdf]

Placebo intervention in general practice

Please read this first:

We define a **placebo intervention** as a diagnostic or therapeutic sham intervention or as an intervention with substances or physical methods which have no direct pharmacological, biochemical or physical mechanism of action according to the current standard of knowledge. The term includes a considerable variety of interventions, thus not only the administration of lactose tablets or isotonic saline solution.

Pure placebos are inert substances or methods such as sugar pills or isotonic saline solution.
Impure placebos refer to substances or methods which have a known pharmacological or physical activity but which cannot be expected to have any direct therapeutic effects for the respective disease and in the chosen dosage, e.g. vitamin infusions for cancer or peppermint pills for pharyngitis.

The questions refer to medical practice and **not** to clinical research.

1. Personal

Age: _____ Gender: □ Female □ Male

How many days per week do you work in your medical office? (rounded up in half days) _____

How many patients do you see per day? □ <16 □ 16-30 □ >30

2. Do you apply placebo interventions to your patients[1]?

If yes, mark all applicable options in the box below.

	... I have already used	... I have not used so far	… is not a placebo intervention.
Positive suggestions	□	□	□
Simple ointments and/or bandages for contusions without visible skin damage	□	□	□
„Sugar pills“	□	□	□
Injections with saline solution	□	□	□
Therapies without pharmacological or physical efficacy for the patient’s conditions (e.g. vitamins or antibiotics without approved indication)	□	□	□
Diagnostic practices, maybe on patient’s request or to calm the patient: • non-essential physical examinations of the patient	□	□	□
• non-essential technical examinations of the patient **without** relevant risks (e.g. ultrasound, MRI)	□	□	□
• non-essential technical examinations of the patient **with** relevant risks (e.g. computer tomography)	□	□	□
Others (please add):	□ □ □	□ □ □	□ □ □

□ I **do not** apply **placebo interventions** to my patients (Please go to **question 7**).

[1] For simplicity we did not adapt the questionnaire to include children’s care, but this is explicitly included.

3. When and why do you use placebo interventions?

	Placebos used *		
	**pure** and impure placebos *	only **impure** placebos *	no placebos at all
To conform with the requests of the patient	□	□	□
To gain a therapeutic advantage through the placebo effect	□	□	□
To still be able to offer a treatment option to a patient with an „incurable“ disease	□	□	□
To offer a treatment in situations in which standard treatments may strongly burden patients with side effects or are contraindicated	□	□	□
To offer a treatment to patients whose complaints and test results are not attributable to a certain disease (unspecific complaints)	□	□	□
To offer treatment to difficult patients with psychological peculiarities, i.e. constant unwarranted complaints	□	□	□
To test whether the pain is psychogenic or organic	□	□	□
To avoid drug addiction	□	□	□
Others (please add):	□<br>□<br>□	□<br>□<br>□	□<br>□<br>□

* Definitions: see box on the first page

4. How often do you prescribe or administer <u>pure</u> placebos, e.g. sugar pills or isotonic saline solution?

(please mark only the one **most** appropriate)	pure placebos
Daily	□
Approximately once a week	□
Approximately once a month	□
More seldom as once a month	□
Never	□

5. If you use <u>pure</u> placebos therapeutically – what do you tell the patient?

I tell that …<br>(please mark only the **one** most appropriate)	
… this is a medication / a therapy.	□
… this is a placebo.	□
… this is a medicine with no specific effect.	□
I say nothing.	□
I **never** give pure placebo.	□

6. If you use i<u>mpure</u> placebos therapeutically – what do you tell the patient?

I tell that …<br>(please mark only the **one** most appropriate)	
… this is a medication / a therapy.	□
… this is a placebo.	□
… this is a medicine with no specific effect.	□
I say nothing.	□
I **never** give impure placebo.	□

7. The use of <u>pure</u> placebos in the medical practice...

	I agree	I am uncertain	I disagree
... must be rejected in principle because it is ineffective.	□	□	□
... must be rejected in principle because it implies deceiving the patient.	□	□	□
… must be rejected in principle because of legal concerns.	□	□	□
... can be used as long as physician and patient work together in partnership.	□	□	□
... is acceptable for the benefit of the patient and for minimizing harm to the patient.	□	□	□
... is for me a traditional component of medical practice.	□	□	□

8. The use of <u>impure</u> placebos in the medical practice...

	I agree	I am uncertain	I disagree
... must be rejected in principle because it is ineffective.	□	□	□
... must be rejected in principle because it implies deceiving the patient.	□	□	□
… must be rejected in principle because of legal concerns.	□	□	□
... can be used as long as physician and patient work together in partnership.	□	□	□
... is acceptable for the benefit of the patient and for minimizing harm to the patient.	□	□	□
... is for me a traditional component of medical practice.	□	□	□

9. Do you think patients would be disappointed if they learned that they have been intentionally treated with a <u>pure</u> placebo?

Yes, many of my patients	□
Yes, a few of my patients	□
Mostly no	□
I do not know	□

10. Do you think patients would be disappointed if they learned that they have been intentionally treated with an <u>impure</u> placebo?

Yes, many of my patients	□
Yes, a few of my patients	□
Mostly no	□
I do not know	□

11. *Training* about placebo:

My medical studies included insufficient training about ...<br>Please mark all appropriate.	
● … results from placebo research	□
● … concepts of placebo (definitions, mode of action)	□
● … the use of placebo in medical practice	□
The information about placebo during my medical studies were **sufficient**	□
Information about placebo is inappropriate in medical studies	□
Further **advanced training** on the topics covered is desirable	□

12. Do you agree with the following statements?

(Please mark only the **one** most appropriate.)	Yes	No	I am uncertain
Placebo interventions initiate **self-healing processes** in the patient	□	□	□
The clinical effects of placebo interventions are mostly **negligibly small**	□	□	□
Almost **every medical intervention** is accompanied by placebo effects	□	□	□
The **percentage of patients who benefit from placebos** is as a rule 30-35%	□	□	□
There are **certain human characteristics that make some people** more responsive to placebos than others	□	□	□
Pure placebos have an effect on **subjective symptoms**	□	□	□
Pure placebos have an effect on both subjective symptoms **and objective signs**	□	□	□
Pure placebos can be used to distinguish between **psychogenic or organic** pain	□	□	□
The oral administration of pure placebos can cause **relevant side effects**	□	□	□
If supposed side effects of remedies occur the tentative administration of placebos can help improve future **compliance**	□	□	□
Do you consider the **„drug“ doctor** important for the success of the treatment?	□	□	□
Which percentage of patients state a clinically relevant decrease of the pain after a placebo injection on the first **postoperative** day after an abdominal surgery?			Approx. ___%

13. Have you undergone a therapy yourself that you know is not scientifically proved?

□ Yes	□ No

14. In your opinion does the mentioned alternative methods of therapy predominantly base on the exploitation of placebo effects? Which of them do you use or prescribe?

Please mark all appropriate.	… is predominantly based on exploitation of placebo effects			I use it or prescribe it
	Yes	No	Don't know	
Acupuncture	□	□	□	□
Homeopathy	□	□	□	□
Hypnosis	□	□	□	□
Neural therapy (therapeutic use of local anaesthetics)	□	□	□	□
Osteopathy	□	□	□	□
Reiki and other forms of laying on of hands	□	□	□	□
TENS (Transcutaneous Electrical Nerve Stimulation)	□	□	□	□

Thank you very much for your participation!

Dr. M. Fässler Dr. M. Gnädinger Prof. Dr. Dr. N. Biller-Andorno
